# Association of screen time and cardiometabolic risk in school-aged children

**DOI:** 10.1016/j.pmedr.2020.101183

**Published:** 2020-08-21

**Authors:** Leigh M. Vanderloo, Charles D.G. Keown-Stoneman, Harunya Sivanesan, Patricia C. Parkin, Jonathon L. Maguire, Laura N. Anderson, Mark S. Tremblay, Catherine S. Birken, on behalf of the TARGet Kids! Collaborative

**Affiliations:** a The Hospital for Sick Children Research Institute, Child Health Evaluative Sciences, Toronto, Ontario, Canada; b The Applied Health Research Centre of the Li Ka Shing Knowledge Institute of St. Michael’s Hospital, Toronto, Ontario, Canada; c Division of Biostatistics, Dalla Lana School of Public Health, University of Toronto, Toronto, Ontario, Canada; d School of Dalla Lana Public Health, Epidemiology, University of Toronto, Toronto, Ontario, Canada; e Division of Pediatric Medicine, Department of Pediatrics, The Hospital for Sick Children, University of Toronto, 555 University Avenue, Toronto, Ontario M5G 1XB, Canada; f Institute of Health Policy, Management and Evaluation, Dalla Lana School of Public Health, University of Toronto, Toronto, Ontario, Canada; g Department of Pediatrics, Faculty of Medicine, University of Toronto, Toronto, Ontario, Canada; h Department of Pediatrics, St. Michael’s Hospital, Toronto, Ontario, Canada; i Department of Health Research Methods, Evidence, and Impact, McMaster University, Hamilton, Canada; j Healthy Active Living and Obesity Research, CHEO Research Institute, Ottawa, Canada

**Keywords:** TV, Computer, Triglycerides, Waist circumference, Systolic blood pressure, Glucose, Cholesterol

## Abstract

Screen use has become a pervasive behaviour among children and has been linked to adverse health outcomes. The objective of this study was to examine the association between screen time and a comprehensive total cardiometabolic risk (CMR) score in school-aged children (7–12-years), as well as individual CMR factors. In this longitudinal study, screen time was measured over time (average duration of follow-up was 17.4 months) via parent-report. Anthropometric measurements, blood pressure, and biospecimens were collected over time and used to calculate CMR score [sum of age and sex standardized z-scores of systolic blood pressure (SBP), glucose, log-triglycerides, waist circumference (WC), and negative high-density lipoprotein cholesterol (HDL-c)/square-root of 5]. Generalized estimating equations (GEE) were used to examine the association between screen time and total CMR score as well as individual CMR factors. A total of 567 children with repeated measures were included. There was no evidence of an association between parent-reported child screen time and total CMR score (adjusted β = −0.01, 95% CI [−0.03, 0.005], 0.16). Screen time was inversely associated HDL-c (adjusted β = −0.008, 95% CI [−0.011, −0.005], *p* = 0.016), but there was no evidence that the other CMR components were associated with screen time. Among children 7–12 years, there was no evidence of an association between parent-reported child screen time and total CMR, but increased screen time was associated with slightly lower HDL-c. Research is needed to understand screen-related contextual factors which may be related to CMR factors.

## Highlights

• No evidence of an association between children’s parental-reported screen time and total cardiometabolic risk score.
• Weak association between increased screen time and reduced HDL cholesterol in children.
• No sex or age interactions detected between parental-reported screen time and cardiometabolic risk.

## 1 Introduction

Childhood exposure to digital media and screen use is increasing, as are concerns regarding *how* screen time affects children’s health. Excessive screen time in school-aged children (5–17 years) has been associated with negative physiological and psychosocial health outcomes. (LeBlanc et al., 2012, Tremblay et al., 2010) This is concerning as 36% of 5- to 9-year-olds (Roberts et al., 2017) and 92% of 10- to 17-year-olds (Janssen and Roberts, 2017) are exceeding current screen time recommendations of ≤2 h of recreational screen time per day. (Sedentary Behaviour, American Academy of Pediatrics, 2016, Ponti, 2019) From a mechanistic perspective, time spent using digital screens may translate into less time spent sleeping and being physical active, as well as more time eating in front of screens and more frequent exposure to unhealthy food and beverage marketing. Today, the length of time children spend with digital media exceeds that of any other activity in which they engage apart from sleeping. (Christakis et al., 2004) As a result of these factors, children today may be increasingly at risk for obesity, cardiovascular disease and related comorbidities. Targeted investigations are needed to further understand the relationship between screen time and cardiometabolic risk (CMR) factors in children.

CMR factors, specifically serum lipids (McNeal et al., 2013) and blood pressure, (Chen, 2008) track from childhood to adulthood, supporting the importance of these measures as early indicators of the atherosclerotic process. (Juhola et al., 2013) While CMR factors are known to exhibit within-person variability, (Gillman and Cook, 1995, Oikonen et al., 2016) improved reliability of such measures can be achieved when they are assessed over multiple instances in children. (Gillman and Cook, 1995, Oikonen et al., 2016) Identifying an association between screen time and CMR factors is important as screen time in childhood is a potentially modifiable target for cardiometabolic disease prevention.

Of the scant research published in this area of screen use and CMR, studies have been limited by small sample sizes, cross-sectional designs, and/or focused on single categories or settings of screen time use. For example, Martinez-Gomez and colleagues examined videogame use among 13-to-17-year olds (Martinez-Gó Mez et al., 2012) and found that console videogames, but not computer games, were positively associated with diastolic blood pressure, mean arterial pressure, triglycerides, and a clustered cardiometabolic risk score. Keane and colleagues accounted for screen time only on weekdays, and found that screen time was associated with an increased risk of overweight/obese. (Keane et al., 2017) To develop intervention targets, data on total screen time (hours/day), device type , and their relationship to CMR factors is required.

The objective of this study was to determine whether screen time was associated with total CMR score among healthy school-aged children (7- to 12-years). Secondary objectives included examining the association between screen time and individual factors of the CMR score among this same age group.

## 2 Methods

### 2.1 Study design and participants

A longitudinal study was conducted using concurrent measures of screen time and CMR measured repeatedly among children participating in The Applied Research Group for Kids (TARGet Kids!) – an open cohort study with ongoing recruitment. TARGet Kids! is a practice-based research network for children in Canada that recruits children from primary care practices in the Greater Toronto Area, Canada. For this study, children were included if they were between the ages of 7–12 and had at least one visit between July 2008 and September 2018. Data from children who had any additional follow-up visits with repeated measures were included, increasing the potential power of the analyses. (Diggle et al., 2002) Children who had incomplete data on screen time or CMR biomarkers, or who had health conditions affecting growth at recruitment (e.g., failure to thrive, cystic fibrosis, severe developmental delays, chronic conditions at enrollment [except asthma]), were excluded from the study. Research Ethics Board approval was obtained from the Hospital for Sick Children and St. Michael’s Hospital and parents of participating children provided written informed consent. TARGet Kids! is registered at www.clinicaltrials.gov; NCT01869530.

### 2.2 Exposure variable – Screen time

The primary exposure was child screen time measured via a parent-completed questionnaire. (Carsley et al., 2015) Using questions based on the Canadian Community Health Survey parents were asked to report the time (minutes) their child spent using TV, DVD/video, computer/laptop, video games, smartphones, and tablets on a typical weekday and weekend day. Total screen time was operationalized by creating a comprehensive weighted average of parental-reported time spent in front of TV, DVD, computer, video game, smartphones, and tablets by weekday and weekend day [(mins on typical week day × 5) + (mins on typical weekend × 2)/7].

### 2.3 Outcome variable – Cardiometabolic risk

The primary outcome was total CMR score, (Eisenmann et al., 2010, Eisenmann, 2008) whereby the lower the score, the lower the cardiometabolic risk. (Kamel et al., 2018) Specifically, Eisenmann’s CMR scoring approach, (Eisenmann, 2008) which has demonstrated strong agreement and construct validity with similarly-aged children, was used. To combine the five CMR factors (waist circumference, high-density lipoprotein cholesterol, systolic blood pressure, log triglycerides and glucose, as well as non– high-density lipoprotein cholesterol), each was internally standardized in the whole TARGet Kids! cohort using age and sex stratified z-scores (subtracting the mean and dividing by the standard deviation). (Sage Research Methods. Z transformation. In: Allen M, ed. The SAGE Encyclopedia of Communication Research Methods. ;, 2017) The total CMR score was then calculated by adding the z-scores of waist circumference, negative high-density lipoprotein cholesterol (i.e., z-score of high-density lipoprotein cholesterol was multiplied by −1 as it is inversely related to the total CMR score), systolic blood pressure, log triglycerides, and glucose, and then dividing the sum by the square-root of five. (Neto et al., 2014, Kelly et al., 2011, Hjorth et al., 2014, Anderson et al., 2016) Secondary outcomes included the individual CMR biomarkers. (Anderson et al., 2016, Chung et al., 2016, Chinapaw et al., 2012, Chinapaw et al., 2014, Taverno Ross et al., 2013, Vaisto et al., 2014)

Height, weight and waist circumference were measured by trained research staff during clinic visits. Standing height was measured using a stadiometer (SECA, Germany), weight was measured using a precision digital scale (SECA, Germany), and waist circumference was measured using standardized protocols with a measuring tape. (Centers for Disease Control and Prevention and National Center for Health Statistics. Third National Health and Nutrition Examination Survey (NHANES III) Anthropometric Procedures. Centers Dis Control Prev Natl Cent Heal Stat. Published online, 2003) Systolic blood pressure was also measured by trained research assistants at clinic visits. An appropriately-sized pediatric cuff on the child’s right upper arm was used to measure systolic blood pressure by auscultation once per visit and after a period of rest. Glucose, high-density lipoprotein cholesterol, non-high-density lipoprotein cholesterol, and triglycerides were measured in non-fasting blood samples (4–7 ml) drawn by trained pediatric phlebotomists using standard guidelines previously described by Anderson et al. (Anderson et al., 2017) and transported to Mount Sinai Laboratory (www.mountsinaiservices.com) for analysis. As collecting fasting blood samples from this population can be difficult, and previous studies have shown that duration of fasting has a minimal impact on glucose and lipid profile in children, non-fasting samples were used. (Kamel et al., 2018, Steiner et al., 2011) Time since last drink (except water) and/or meal was recorded during blood collection and adjusted for in the analysis. Concentrations of glucose were measured using an enzymatic reference method with hexokinase; triglycerides, high-density lipoprotein cholesterol, and non-high-density lipoprotein cholesterol were measured using the enzymatic colorimetric method on the Roche Modular platform (Roche Diagnostics, Laval, Canada).

### 2.4 Other variables – Covariates

Confounding variables were identified *a priori* based on previous literature and were collected via the aforementioned parent-reported child health questionnaire. (Carson et al., 2017, Timmons et al., 2012, Poitras et al., 2016) Specifically, models were adjusted for age, sex, fasting time (for waist circumference, glucose, HDL-c, triglycerides), height (for systolic blood pressure), maternal education, maternal ethnicity, family history of cardiovascular disease, and annual household income.

### 2.5 Statistical analyses

All statistical analyses were carried out using R (version 3.4.3). (Core and Team, 2017) Data were cleaned and outliers removed based on biological implausibility as recommended by current standards; (Plumptre et al., 2017, Littman et al., 2012, Qiao et al., 2000, Gardner et al., 2000, Palaniappan et al., 2003) specifically, for systolic blood pressure, we used the literature to identify the thresholds (>0 or > 200 mmHg). For the other CMR components (BMI, waist circumference, glucose, HDL cholesterol, non-HDL cholesterol), we excluded all the values that are outside range of −6 and 6 for z-scores of waist circumference, glucose, HDL cholesterol, and non-HDL cholesterol. All outcome variables were assessed for approximate normality of distribution and any necessary transformations were performed. To address a skewed distribution for the triglyceride variable, a log-transformation was completed.

Scaled to a unit increase of 60 min, Gaussian generalized estimating equations (GEE) were used to examine the association between screen time (using restricted cubic splines with 4 knots) and total CMR, as well as the individual CMR factors, while accounting for within-subject repeated measures using an exchangeable correlation structure. Multiple imputation was used for missing covariate data (difference between imputed and non-imputed data > 0.05). Models were run on 10 imputed datasets using the ‘mice’ package in R. (van Buuren and Groothuis-Oudshoorn, 2011) The proportion of missing values for all covariates was < 10%. All models were adjusted for covariates, selected *a priori* based on the literature, (Martinez-Gó Mez et al., 2012, Keane et al., 2017, Chinapaw et al., 2014, Taverno Ross et al., 2013, Vaisto et al., 2014, Bucksch et al., 2016, Lee et al., 2017, Altenburg et al., 2012, Huang et al., 2016, Aranha Crispim et al., 2014) and collected at baseline from a parent-reported questionnaire: child age and sex, maternal ethnicity, parental income, family history of cardiovascular disease, fasting time, and physical activity (habitual as reported by parents). Glucose, triglycerides, and cholesterol were further adjusted for fasting hours, and blood pressure was also adjusted for child’s height. Due to the possibility of child’s body mass index being on the causal pathway between screen use and CMR outcomes, this variable was not included in the models as a means of unconfounding this possible association. Sex-screen time, age-screen, and year-screen time interactions were also tested in the model to determine whether associations between screen time and CMR differed by sex, age, or year (chronological year).

For the secondary objective, logistic GEE regression models, using repeated measures from multiple visits and scaled to a unit increase of 60 min), were run to determine whether higher screen time (using restricted cubic splines with 4 knots) was associated with dichotomous measures of each CMR factor using published cut-points, where available: higher waist circumference (>90th percentile), high-density lipoprotein cholesterol (<1.17 mmol/L), systolic blood pressure (≥90^th^ percentile) (Flynn et al., 2017), triglycerides (>0.84 mmol/L), glucose (>90th percentile), and non-high-density lipoprotein cholesterol (>3.11 mmol/L). To verify assumptions of the regression models were met, (Warner, 2008, Logistic and Diagnostics, 1981, Hosmer and Lemeshow, 1980) residual analyses were performed for both Gaussian and logistic regression. Based on variance inflation factors (VIF), (Murray et al., 2012) there was no evidence of multi-collinearity in any of the models for either the Gaussian or logistic regression analyses (data available upon request). Sensitivity analyses were also performed using only complete cases reporting all screen time variables.

## 3 Results

**Fig. 1:**
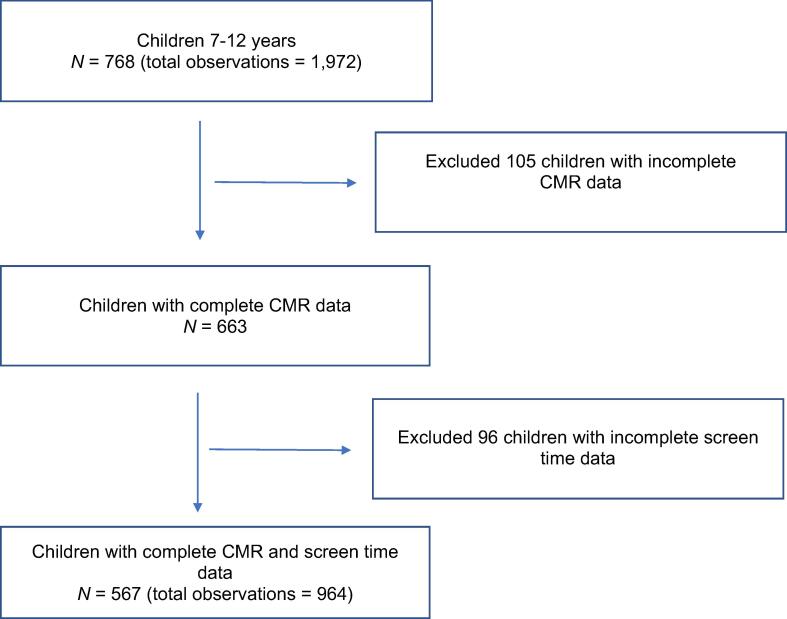
Participant flow chart.

A total of 567 participants were retained for analyses (Fig. 1). Baseline descriptive characteristics of children included in this study are presented in Table 1. The average age of participants at baseline was 7.8 years (or 93.7 ± 16.7 months), 44.7% were female, and 72.1% of respondents reported European ethnicity. Among these children, 388 (68%) had 2 and 9 (2%) had 3 or more concurrent measures of screen time and CMR over multiple visits, respectively, resulting in 964 observations available for analysis. On average, participants spent 1287 (*SD* = 271) min/wk (or 183.86 mins/day) using screens (TV, video games, smartphones, tablets). Children spent most time in front of a TV compared to other types of screens, such as computers or smartphones. See Table 2 for additional details.

**Table 1:** Sample descriptive characteristics at baseline (*n* = 567).

Variable	Mean ± SD	N (%)	Percent missing
Child age (months)	93.7 ± 16.7		1.9%
Child Sex			0.00%
Female		431 (44.7%)	–
Male		533 (55.3%)	–
Maternal ethnicity			5.8%
East Asian		93 (9.6%)	–
European		695 (72.1%)	–
South/Southeast Asian		56 (5.8%)	–
Other		120 (12.4%)	–
Parental annual income			8.2%
<$30.000		24 (4.2%)	–
$30,000 to $79,999		102 (18.0%)	–
$80,000 to $149,000		181 (32.0%)	–
$150,000 +		260 (45.8%)	–
Family history of CVD			7.5%
Yes		158 (16.4%)	–
No		806 (83.6%)	–
Average duration of follow-up	17.4 ± 5.7		
Child’s height (cm)	124.8 ± 13.1		2.5%
Fasting time (hour)	2.4 ± 0.9		3.5%
Total screen time (min/wk)^a^	1,287 ± 271		–
Glucose (mmol/L)	4.7 ± 1.4		–
SBP (mmHg)	92 ± 39		–
Triglycerides (mmol/L)	−0.961 + 0.04		–
HDL cholesterol (mmol/L)	1.51 ± 0.89		–
Waist circumference (cm)	59.5 ± 12.7		–
Non-HDL cholesterol (mmol/L)	2.5 ± 0.9		–
Total CMR (z-score)	−0.04 ± 1.18		–
Physical activity (mins/day)	44 ± 13		–

***Notes***. CMR: cardiometabolic risk; SBP: systolic blood pressure; HDL: high-density lipoprotein cholesterol; BMI: body mass index; WC: waist circumference; CVD: cardiovascular disease; SSB: sugar-sweetened beverages. Continuous variables are shown as median, quartiles, and mean/standard deviation. Categorical variables are shown as number of subjects and percentages.

a Total Screen Time was calculated as the sum of: *Weekday screen time use* (sum of TV, DVD, computer, videogame, smartphones, and tablets use on a typical weekday), *Weekend day screen time use* (sum of TV, DVD, computer, videogame, smartphones, and tablets use on a typical weekend day).

**Table 2:** Screen time (minutes/day) by screen type (TV, DVD, videogame, computer, smartphones, tablets), and by weekday/weekend day.

Variables	Mean ± SD
*Weekday screen use*^a^	
TV	48 ± 1.0
DVD	16.2 ± 0.5
Videogame	12 ± 0.2
Computer	6 ± 0.3
Smartphone and tablet	72 ± 0.4

*Weekend day screen use*^b^	
TV	114 ± 1.1
DVD	24 ± 0.7
Videogame	30 ± 0.6
Computer	42 ± 0.3
Smartphone and tablet	48 ± 0.6

Notes:

a Weekday screen time use includes the sum of TV, DVD, computer, videogame, smartphones, and tablets device use from a typical weekday.

b Weekend day screen time use includes the sum of TV, DVD, computer, videogame, smartphones, and tablets use from a typical weekend day.

### 3.1 Parent-Reported child screen time and cardiometabolic risk in School-Aged children

For the primary analysis, adjusted GEEs indicated no evidence of an association between parent-reported child screen time and total CMR score (β = −0.01, 95% CI [−0.03, 0.005, *p* = 0.16). Evidence of an association, though very small, was identified at the 5% level of significance between screen time and high-density lipoprotein cholesterol (β = −0.008, 95% CI [−0.011, −0.005], *p* = 0.02). It was estimated that every additional 60 min of screen time was associated with a 0.008 mmol/l decrease in high-density lipoprotein cholesterol.

For the secondary objective, analyses were conducted for each CMR factor using established cut-points. There was no evidence that the remaining individual CMR factors (i.e., adjusted: glucose [*p* = 0.53], waist circumference [*p* = 0.82], non-high-density lipoprotein cholesterol [*p* = 0.30], systolic blood pressure [*p* = 0.65], triglycerides [*p* = 0.15]) were associated with screen time (Table 3). There was no evidence of an interaction between age, sex, or year (*p* > 0.05). Adjusted logistic GEEs revealed no evidence of an association between screen time and high-risk cut-offs for any of the individual CMR markers (*p* > 0.05), including high-density lipoprotein cholesterol (Table 4). In the planned sensitivity analyses, results from children with complete screen time data were similar to those obtained from children with incomplete screen time data *n* = 96; *p* = 0.79; 95% CI −0.21, 0.56).

**Table 3:** Gaussian GEE model for association between screen time and total cardiometabolic risk score and individuals risk factors (*n* = 567 participants) – per increase of 60 min of screen time.

Unadjusted model			Adjusted model^a^	
Outcome variable	B (95% CI)	*p*	B (95% CI)	*p*
Total cardiometabolic risk score	0.01 (−0.03, 0.008)	0.27	−0.01 (−0.03, 0.005)	0.16
Glucose^b^	−0.005 (−0.02, 0.008)	0.51	−0.004 (−0.02, 0.008)	0.53
Systolic blood pressure^b^	−0.004 (−0.02, 0.01)	0.58	−0.004 (−0.03, 0.02)	0.65
Triglycerides (log*-transformed*)^b^	−0.008 (−0.02, 0.005)	0.21	−0.010 (−0.02, 0.004)	0.15
High-density lipoprotein (*inversed*)^b^	0.009 (−0.005, 0.02)	0.20	**0.008 (0.005, 0.011)**	**0.02**
Waist circumference	0.0006 (−0.01, 0.01)	0.92	−0.001 (−0.01, 0.01)	0.82
Non-high-density lipoprotein^b^	−0.01 (−0.03, 0.0005)	0.06	−0.02 (−0.03, 0.002)	0.30

Notes.

All variables are z-transformed (i.e., 1−unit increase/decrease = change of 1SD).

VIF for the model = 3.1.

a Adjusted model includes adjustment for child age, child sex, maternal ethnicity, parental income, family history of CVD, and physical activity.

b Model with systolic blood pressure is further adjusted for height. Glucose, cholesterol, and triglycerides were adjusted for fasting time.

**Table 4:** Logistic GEE model for association between screen time and cardiometabolic risk factors (*n* = 567 participants) – per increase of 60 min of screen time.

Unadjusted model				Adjusted model^a^	
Variable	Cut-points for high-risk	OR (95% CI)	*p*	OR (95% CI)	*p*
Glucose (mmol/L)	>90th percentile	1.05 (1.07, 1.18)	0.41	0.99 (0.95, 1.04)	0.88
Systolic blood pressure (mmHg)^b^	>90th percentile	0.96 (0.86, 1.08)	0.51	1.01 (0.97, 1.05)	0.67
Triglycerides (mmol/L) (log*-transformed*)^b^	>0.84 mmol/L	1.03 (0.90, 1.17)	0.67	1.04 (0.70, 1.54)	0.85
High-density lipoprotein (mmol/L) (*inversed*)^b^	<1.17 mmol/L	0.95 (0.85, 1.06)	0.38	0.98 (0.93, 1.03)	0.37
Waist circumference (cm)	>90th percentile	1.03 (0.92, 1.15)	0.66	0.972 (0.93, 1.02)	0.21
Non-high-density lipoprotein (mmol/L)^b^	>3.11 mmol/L	0.85 (0.62,1.69)	0.31	0.66 (0.40, 1.10)	0.11

Notes:

Guidelines by Flynn et al. (2017) and Expert Panel on Integrated Guidelines for Cardiovascular Health and Risk Reduction in Children and Adolescents (2011) were used to determine elevated risk (cut-offs) for individual cardiometabolic factors.

VIF for the model = 2.7.

a Adjusted model includes adjustment for child age, child sex, maternal ethnicity, parental income, family history of CVD, and physical activity.

b Model with systolic blood pressure is further adjusted for height. Glucose, cholesterol, and triglycerides were adjusted for fasting time.

## 4 Discussion

In this study of 567 school-aged children (7–12 years), it was reported that participants spent approximately three hours per day engaged in screen-based pursuits. Compared to parent-reported child screen time, national surveys report Canadian participants (5–11 years) spending 2.5 h using screens per day. (Statistics Canada. Physical activity and screen time among Canadian children and youth, 2016) American children (9–11 years) self-reported spending 3.6 h of screen time per day, (Walsh et al., 2018) whereas, Dutch and Hungarian participants (10–12 years) self-described 116 ± 64 min per day watching TV and 85 ± 57 min per day using the computer. (Chinapaw et al., 2012).

No evidence of an association between parent-reported child screen time and total CMR score was identified, and no effect modification was observed by age or sex. Given the past noted underlying patterns and structures among the included cardiometabolic variables (e.g., glucose, lipids, blood pressure, and waist circumference), (Lambert et al., 2004, Chen et al., 1999, Moreno et al., 2002) these findings were unexpected. However, the use of subjective screen time data coupled with the fact that the timeframe to determine an association between screen time and children’s cardiometabolic health in the present study may be too short, may help explain the lack of significant findings.

Findings from this study are similar to Rey-Lopez et al.’s cross-sectional (*n* = 796, age 12.5–17.5 years) study which reported that self-reported TV-viewing was not related to metabolic risk in either sex, (Rey-López et al., 2013) and cross-sectional work by Chinapaw et al. (*n* = 142, age 10–13 years) who identified no association between parent-reported TV or computer time and metabolic indicators after adjusting for gender, country, and physical activity. (Chinapaw et al., 2014) Altenburg et al. (*n* = 125, age 12–18 years) also found no association between self-reported total screen time (TV viewing and computer time) and clustered CMR score or individual CMR factors. (Altenburg et al., 2012) Vaisto et al. observed that higher levels of parented-reported TV-viewing were related to a comprehensive CMR score. (Vaisto et al., 2014) The current study extends the work by Vaisto et al. by incorporating repeated measures, which results in increased power (compared to a simple cross-sectional approach with the same sample size). In the current study, additional covariates (i.e., physical activity and ethnicity) were included as confounders. (Vaisto et al., 2014) It is also worth noting that because the present study’s analytical approach modelled a 60-minute increase in screen time, it does not allow for the selective reallocation of this time. For example, if the 60-minute increase in screen time was replaced with 60 min of higher-intensity physical activity (like moderate-to-vigorous physical activity [MVPA]), it is possible that more meaningful differences may have emerged (manifested by the combined benefits of reduced screen time and increased MVPA). Conversely, replacing screen time with an alternate form of sedentary behaviour may have elicited stronger associations.

While no evidence of an association between screen time and total CMR score was observed, evidence of a very small effect was ascertained between screen time and an individual cardiometabolic marker was identified. Specifically, higher screen time was associated with slightly lower high-density lipoprotein cholesterol. To provide a clinically meaningful interpretation of this effect size and to compare effect estimates across age periods, it can be inferred that every 60-minute increase in screen time translate to an 0028 mmol/L decrease in high-density lipoprotein cholesterol for an average 7-year-old boy, for instance. In other studies, Altenburg et al. found that computer time was associated with increased total cholesterol and LDL-c. Although a slightly older sample, Martinez-Gomez et al. identified that increased video game use (self-reported) was associated with decreased triglycerides, (Martinez-Gó Mez et al., 2012) but not total CMR, among 13–17-year-olds. Based on most of the published work to date, while evidence of an association between screen time and total CMR and lipid outcomes are unclear, with two studies identifying associations in the opposite direction expected. The clinical significance of these findings with very small effect sizes remains uncertain.

No evidence of sex or age interactions between screen time and CMR were identified, consistent with other published work. (Keane et al., 2017, Chinapaw et al., 2014) However, Rey-Lopez and colleagues found that the clustering of CMR differed in boys (compared to girls) when playing videogames >4 h/day. (Rey-López et al., 2013) Vaisto et al. (*n* = 468, age 6–8 years) also reported contrasting findings, (Vaisto et al., 2014) with young males having better CMR biomarkers than their female counterparts in relation to sedentary behaviours (screens).

While the results of a published review reported that CMR risk factor clustering is stable from childhood into adulthood, (Camhi and Katzmarzyk, 2010) it is unclear if the relationship between screen time and CMR change as children age. Additional confirmatory studies may be required to examine whether associations between screen time and CMR change over childhood through to adolescence and adulthood, given the likely changes in frequency and type of screen time use as children age. It is possible that the inherent cardiometabolic health of children of this age masks an underlying association of screen time on cardiometabolic health, such that only longer-term longitudinal studies will detect the impact.

### 4.1 Strengths and Limitations

The primary strength of this study was the use of repeated measures for both the exposure and outcome variables in a relatively large sample of children, thus reducing within-person variability and increasing statistical power (and precision). (Gillman and Cook, 1995, Marcovina et al., 1994) However, this factor is hindered by the fact that 30% of the sample had only one measure, thus resulting in an ‘unbalanced’ longitudinal study. An additional strength was the use of a more comprehensive screen time variable – various devices (TV, DVD/video, computer/laptop, smartphones, tablets) and on weekdays and weekends. Limitations include the use of parent-reported data, which tends to underestimate children’s screen time, (Association and Underestimate, 2014) although a directly/objective measure of screen time does not currently exist. Further, the authors were unable to account for multi-screen use among (e.g., children watching TV while using their phone or a tablet), therefore potentially confounding the intersectionality across different screens used in a particular sitting– only data on screen use duration and types were collected. Given the propensity for young children to utilize multiple screens at once, future research should examine these associations among children and youth, particularly with regards to their cardiometabolic health. Although the use of a continuous CMR score has been shown to be predictive of subclinical atherosclerosis in older children, (Magnussen et al., 2010) the lack of agreed upon cut-points or thresholds coupled with the existence of various definitions have been used to define the CMR score, (Kamel et al., 2018) may present as a limitation when comparing effect sizes across other studies. Generalizability of the findings from this work are limited as many participants were from families with annual incomes above $80,000 (~80%), of European decent (72.1%), and from the greater Toronto area. However, the distribution of ethnicity in the present study was comparable to national census data. ([66]) Children with incomplete CMR data were excluded; however, no significant differences between the sample of children with and without blood work was reported (data available upon request). Future analyses may also consider adjusting for practice site to account for any differences.

## 5 Conclusion

Results from this study found no evidence that parent-reported child screen time was associated with total CMR among children 7–12 years, but screen time was associated with slightly lower HDL-c. While there is good evidence to suggest that the reduction of screen time has many positive impacts on children’s health, like improved cognitive outcomes or associations with less depressive symptoms, (Carson et al., 2017, Janssen and LeBlanc, 2010) based on the present findings of this study, there may not be a strong association between parent-reported child screen time and CMR in school aged children; this finding would be strengthened by valid measures of screen time, including screen-related contextual factors (e.g., various characteristics of children’s physical [location in the home] and social [alone or with siblings or parents] environments), repeated throughout childhood into adolescence and beyond.

## CRediT authorship contribution statement

**Leigh M. Vanderloo:** Conceptualization. **Charles D.G. Keown-Stoneman:** . **Harunya Sivanesan:** . **Patricia C. Parkin:** . **Jonathon L. Maguire:** Conceptualization. **Laura N. Anderson:** . **Mark S. Tremblay:** Conceptualization. **Catherine S. Birken:** Conceptualization.

## References

[b0005] LeBlanc A.G., Spence J.C., Carson V., Connor G.S., Dillman C., Janssen I. (2012). Systematic review of sedentary behaviour and health indicators in the early years (aged 0–4 years). Appl. Physiol. Nutr. Metab..

[b0010] Tremblay M.S., Colley R., Saunders T.J., Healy G.N., Owen N. (2010). Physiological and health implications of a sedentary lifestyle. Appl. Physiol. Nutr. Metab..

[b0015] Roberts K.C., Yao X., Carson V., Chaput J.P., Janssen I.T.M. (2017). Meeting the canadian 24-hour movement guidelines for children and youth. Heal Reports.

[b0020] Janssen I., Roberts K.C.T.W. (2017). Adherence to the 24-Hour Movement Guidelines among 10- to 17-year-old Canadians. Heal Promot. Chronic Dis. Prev. Canada.

[b0025] Sedentary Behaviour, and Sleep. 2016 csepguidelines.ca/wp-content/themes/%0Acsep2017/pdf/Canadian24HourMovementGuidelines2016.pdf.

[b0030] American Academy of Pediatrics - Council on Communications and Media. Media Use in School-Aged Children and Adolescents. Pediatrics. 2016;138(5). 10.1542/peds.2016-2592 27940794

[b0035] Ponti M. (2019). DHTF. Digital Media: Promoting healthy screen use in school-aged. Child. Adolescents.

[b0040] Christakis D.A., Ebel B.E., Rivara F.P.Z.F. (2004). Television, video, and computer game usage in children under 11 years of age. J. Pediatr..

[b0045] McNeal C.J., Underland L., Wilson D.P., Blackett P.R. (2013). Pediatric lipid screening. Clin. Lipidol..

[b0050] Chen X.W.Y. (2008). Tracking of blood pressure from childhood to adulthood: A systematic review and meta-regression analysis. Circulation.

[b0055] Juhola J., Magnussen C.G., Berenson G.S., Venn A., Burns T.L.S.M. (2013). Combined effects of child and adult elevated blood pressure on subclinical atherosclerosis: The International Childhood Cardiovascular Cohort Consortium. Circulation.

[b0060] Gillman M.W., Cook N.R. (1995). Blood pressure measurement in childhood epidemiological studies. Circulation.

[b0065] Oikonen M., Nuotio J., Magnussen C.G., Viikari J.S., Taittonen L., Laitinen T. (2016). Repeated blood pressure measurements in childhood in prediction of hypertension in adulthood. Hypertension.

[b0070] Martinez-Gó Mez D., Gomez-Martinez S., Ruiz J.R., Ortega F.B., Marcos A., Veiga O.L. (2012). Video game playing time and cardiometabolic risk in adolescents: The AFINOS study. Med. Clin. (Barc)..

[b0075] Keane E., Li X., Harrington J.M., Fitzgerald A.P., Perry I.J., Kearney P.M. (2017). Physical activity, sedentary behavior and the risk of overweight and obesity in school-aged children. Pediatr. Exerc. Sci..

[b0080] Diggle P.J., Heagerty P.J., Liang K.-Y.-Z.-S. (2002). Analysis of Longitudinal Data.

[b0085] Carsley S., Borkhoff C.M., Maguire J.L. (2015). Cohort profile: The applied research group for kids (TARGet Kids!). Int. J. Epidemiol..

[b0090] StatsCan. Canadian Community Health Survey.

[b0095] Eisenmann J.C., Laurson K.R., DuBose K.D., Smith B.K., Donnelly J.E. (2010). Construct validity of a continuous metabolic syndrome score in children. Diabetol. Metab. Syndr..

[b0100] Eisenmann J.C. (2008). On the use of a continuous metabolic syndrome score in pediatric research. Cardiovasc. Diabetol..

[b0105] Kamel M., Smith B., Birken W.G., Anderson C.S.L. (2018). Continuous cardiometabolic risk score definitions in early childhood: a scoping review.

[b0110] Sage Research Methods. Z transformation. In: Allen M, ed. The SAGE Encyclopedia of Communication Research Methods. ; 2017.

[b0115] Neto Antonio Stabelini, Wagner de Campos dos S and OMJ G.C., Stabelini Neto A., de Campos W., Dos Santos G.C., Mazzardo Junior O. (2014;14:42.). Metabolic syndrome risk score and time expended in moderate to vigorous physical activity in adolescents. BMC Pediatr..

[b0120] Kelly A.S., Steinberger J., Jacobs D.R., Hong C.P., Moran A., Sinaiko A.R. (2011). Predicting cardiovascular risk in young adulthood from the metabolic syndrome, its component risk factors, and a cluster score in childhood. Int. J. Pediatr. Obes..

[b0125] Hjorth M.F., Chaput J.-P., Damsgaard C.T. (2014). Low physical activity level and short sleep duration are associated with an increased cardio-metabolic risk profile: a longitudinal study in 8–11 year old Danish children. PLoS One.

[b0130] Anderson L.N., Lebovic G., Hamilton J. (2016). Body mass index, waist circumference, and the clustering of cardiometabolic risk factors in early childhood. Paediatr. Perinat. Epidemiol..

[b0135] Chung I.H., Park S., Park M.J., Yoo E.G. (2016). Waist-to-height ratio as an index for cardiometabolic risk in adolescents: Results from the 1998–2008 KNHANES. Yonsei Med. J..

[b0140] Chinapaw M.J.M., Yildirim M., Altenburg T.M. (2012). Objective and self-rated sedentary time and indicators of metabolic health in Dutch and Hungarian 10–12 year olds: The energy-project. PLoS One.

[b0145] Chinapaw M.J.M., Altenburg T.M., van Eijsden M., Gemke R.J.B.J., Vrijkotte T.G.M. (2014). Screen time and cardiometabolic function in Dutch 5–6 year olds: cross-sectional analysis of the ABCD-study. BMC Public Health..

[b0150] Taverno Ross S., Dowda M., Saunders R., Pate R. (2013). Double dose: The cumulative effect of TV viewing at home and in preschool on children’s activity patterns and weight status. Pediatr. Exerc. Sci..

[b0155] Vaisto J., Eloranta A.M., Viitasalo A. (2014). Physical activity and sedentary behaviour in relation to cardiometabolic risk in children: Cross-sectional findings from the Physical Activity and Nutrition in Children (PANIC) Study. Int. J. Behav. Nutr. Phys. Act..

[b0160] Centers for Disease Control and Prevention and National Center for Health Statistics. Third National Health and Nutrition Examination Survey (NHANES III) Anthropometric Procedures. Centers Dis Control Prev Natl Cent Heal Stat. Published online 2003.

[b0165] Anderson L.N., Maguire J.L., Lebovic G. (2017). Duration of fasting, serum lipids, and metabolic profile in early childhood. J. Pediatr..

[b0170] Steiner M.J., Skinner A.C., Perrin E.M. (2011). Fasting might not be necessary before lipid screening: A nationally representative cross-sectional study. Pediatrics.

[b0175] Carson V., Lee E.-Y., Hewitt L. (2017). Systematic review of the relationships between physical activity and health indicators in the early years (0–4 years). BMC Public Health..

[b0180] Timmons B.W., LeBlanc A.G., Carson V., Connor G.S., Dillman C., Janssen I. (2012). Systematic review of physical activity and health in the early years (aged 0–4 years). Appl. Physiol. Nutr. Metab..

[b0185] Poitras V.J., Gray C.E., Borghese M.M. (2016). Systematic review of the relationships between objectively measured physical activity and health indicators in..

[b0190] Core R., Team R. (2017). A language and environment for statistical computing. Published Online.

[b0195] Plumptre L., Anderson L.N., Chen Y. (2017). Longitudinal analysis of sleep duration and cardiometabolic risk in young children. Child Obes.

[b0200] Littman A., Boyko E., McDonell M., Fihn S. (2012). Evaluation of a weight management program for veterans. Prev. Chronic Dis. Published online.

[b0205] Qiao Q., Nakagami T., Tuomilehto J. (2000). Comparison of the fasting and the 2-h glucose criteria for diabetes in different Asian cohorts. Diabetologia..

[b0210] Gardner C., Winkleby M., Fortmann S. (2000). Population frequency distribution of non–high-density lipoprotein cholesterol (third national health and nutrition examination survey [NHANES iii], 1988–1994). Am. J. Cardiol..

[b0215] Palaniappan L, Carnethon M, Fortmann SP. Association between Microalbuminuria and the Metabolic Syndrome: NHANES III. Am J Hypertens. 2003;16(11 I):952-958. doi:10.1016/S0895-7061(03)01009-4. 10.1016/s0895-7061(03)01009-4 14573334

[b0225] van Buuren S., Groothuis-Oudshoorn K. (2011). MICE: Multivariate Imputation by Chained Equations in R. J. Stat. Softw..

[b0230] Bucksch J., Sigmundova D., Hamrik Z. (2016). International trends in adolescent screen-time behaviors from 2002 to 2010. J. Adolesc. Heal..

[b0235] Lee E., Hesketh K., Hunter S. (2017). Meeting new Canadian 24-hour movement guidelines for the early years and associations with adiposity among toddlers living in Edmonton, Canada. BMC Public Health..

[b0240] Altenburg T.M., Hofsteenge G.H., Weijs P.J.M., Delemarre-van de Waal H.A., Chinapaw M.J.M. (2012). Self-reported screen time and cardiometabolic risk in obese dutch adolescents. PLoS One.

[b0245] Huang W.Y., Wong S.H.-S., He G., Salmon J. (2016). Isotemporal substitution analysis for sedentary behavior and body mass index. Med. Sci. Sports Exerc..

[b0250] Aranha Crispim P.A., Gondim Peixoto M.do R., Brandao Veiga Jardim P.C. (2014). Risk factors associated with high blood pressure in two- to five-year-old children. Arq. Bras. Cardiol..

[b0255] Flynn J.T., Kaelber D.C., Baker-Smith C.M., Blowey D., Carroll A.E.D.S. (2017). Expert Panel on Integrated Guidelines for Cardiovascular Health and Risk Reduction in Children and Adolescents: Summary Report. Pediatrics..

[b0260] Warner P. (2008). Ordinal logistic regression. J. Fam Plan Reprod Heal Care. Published online.

[b0265] Pregibon D. (1981). Logistic regression diagnostics. Ann. Stat. Published online.

[b0270] Hosmer D.W., Lemeshow S. (1980). Goodness of fit tests for the multiple logistic regression model. *Commun Stat - Theory Methods*. Published online.

[b0275] Murray L., Nguyen H., Lee Y.-F., Remmenga M.D., Smith D.W. (2012). Variance Inflation Factors in regression models with dummy variables. *Annu. Conf. Appl. Stat. Agric*. Published online.

[b0280] Statistics Canada. Physical activity and screen time among Canadian children and youth, 2016 and 2017. Health Fact Sheets. Published 2019. Accessed April 6, 2020. https://www150.statcan.gc.ca/n1/pub/82-625-x/2019001/article/00003-eng.htm.

[b0285] Walsh J.J., Barnes J.D., Cameron J.D. (2018). Associations between 24 hour movement behaviours and global cognition in US children: a cross-sectional observational study. Lancet. Child. Adolesc. Heal..

[b0290] Lambert M., Paradis G., O’Loughlin J., Delvin E.E., LE Hanley J.A. (2004). Insulin resistance syndrome in a representative sample of children and adolescents from Quebec, Canada. Int. J. Obes. Relat. Metab. Disord..

[b0295] Chen W., Srinivasan S.R., Elkasabany A.B.G. (1999). Cardiovascular risk factors clustering features of insulin resistance syndrome (Syndrome X) in a biracial (Black-White) population of children, adolescents, and young adults: the Bogalusa Heart Study. Am J Epidemiol..

[b0300] Moreno L.A., Pineda I., Rodriguez G., Fleta J., Giner A., Juste M.G.S. (2002). Leptin and metabolic syndrome in obese and non-obese children. 34:3. Horm. Metab. Res..

[b0305] Rey-López J.P., Bel-Serrat S., Santaliestra-Pasías A. (2013). Sedentary behaviour and clustered metabolic risk in adolescents: The HELENA study. Nutr. Metab. Cardiovasc. Dis..

[b0310] Camhi S.M., Katzmarzyk P.T. (2010). Tracking of cardiometabolic risk factor clustering from childhood to adulthood. Int. J. Pediatr. Obes..

[b0315] Marcovina S.M., Gaur V.P., Albers J.J. (1994). Biological variability of cholesterol, triglyceride, low- and high-density lipoprotein cholesterol, lipoprotein(a), and apolipoproteins A-I and B. Clin. Chem..

[b0320] American Optometric Association. Survey Reveals Parents Drastically Underestimate the Time Kids Spend on Electronic Devices. Published 2014. https://www.aoa.org/newsroom/survey-reveals-parents-drastically-underestimate-the-time-kids-spend-on-electronic-devices.

[b0325] Magnussen C.G., Koskinen J., Chen W., Thomson R., Schmidt M.D., Srinivasan S.R., Kivimaki M., Mattsson N., Kahonen M. (2010). Pediatric metabolic syndrome predicts adulthood metabolic syndrome, subclinical atherosclerosis, and type 2 diabetes mellitus but is no better than body mass index alone: the Bogalusa Heart Study and the Cardiovascular Risk in Young Finns Study. Circulation..

[66] (2016). Census Ontario and Canada -Ethnic Origin..

[b0335] Janssen I., LeBlanc A.G. (2010). Systematic review of the health benefits of physical activity and fitness in school-aged children and youth. Int. J. Behav. Nutr. Phys. Act..

